# Impact of a poor functional capacity on the clinical outcomes in patients with a pacemaker implantation –Results from the Japanese Heart Rhythm Society Registry –

**DOI:** 10.1002/joa3.12459

**Published:** 2020-12-09

**Authors:** Takanori Arimoto, Eiichi Watanabe, Ritsuko Kohno, Kenji Shimeno, Kan Kikuchi, Atsushi Doi, Kanki Inoue, Takashi Nitta, Akihiko Nogami, Haruhiko Abe, Ken Okumura

**Affiliations:** ^1^ Department of Cardiology Yamagata University School of Medicine Yamagata Japan; ^2^ Department of Cardiology Fujita Health University Bantane Hospital Nagoya Japan; ^3^ Department of Heart Rhythm Management University of Occupational and Environmental Health Kitakyushu Japan; ^4^ Department of Cardiology Osaka City General Hospital Osaka Japan; ^5^ Division of Cardiology Japan Community Healthcare Organization Kyushu Hospital Kitakyushu Japan; ^6^ Department of Cardiovascular Medicine Osaka City University Graduate School of Medicine Osaka Japan; ^7^ Division of Cardiology Sakakibara Heart Institute Tokyo Japan; ^8^ Cardiovascular Surgery Nippon Medical School Tokyo Japan; ^9^ Department of Cardiology Faculty of Medicine University of Tsukuba Tsukuba Japan; ^10^ Division of Cardiology Saiseikai Kumamoto Hospital Kumamoto Japan

**Keywords:** functional capacity, pacemaker, prognosis

## Abstract

**Background:**

Functional capacity (FC) correlates with mortality in various cardiovascular diseases. The aim of this study was to examine whether cardiac pacemaker implantations improve the FC and affect the prognosis.

**Methods and Results:**

We prospectively enrolled 621 de novo pacemaker recipients (age 76 ± 9 years, 50.7% male). The FC was assessed by metabolic equivalents (METs) during the implantation and periodically thereafter. The patients were a priori classified into poor FC (<2 METs, n = 40), moderate FC (2 ≤ METs < 4, n = 239), and good FC (≥4 METs, n = 342). Three months after the pacemaker implantation, poor FC or moderate FC patients improved to a good FC by 43%. The distribution of the three FCs remained at those levels until after 1 year of follow‐up (*P* = .18). During a median follow‐up of 2.4 years, 71 patients (11%) had cardiovascular hospitalizations and 35 (5.6%) all‐cause death. A multivariate Cox analysis revealed that a poor FC at baseline was an independent predictor of both cardiovascular hospitalization (hazard ratio [HR] 2.494, *P* = .012) and all‐cause death (HR 3.338, *P* = .016). One year after the pacemaker implantation, the eight who remained with a poor FC had a high mortality rate of 37.5% (*P* < .01).

**Conclusion:**

Approximately half of the poor or moderate FC patients improved to good FC 3 months after the pacemaker implantation. The baseline FC predicted the prognosis, and patients with an improved FC after the pacemaker implantation had a better prognosis.

## Graphical abstract

We examined the temporal trends in the functional capacity (FC) and the relationship between the FC and prognosis in patients receiving an initial pacemaker implantation. The major findings of this study were that (i) 43% of the patients with a poor or moderate FC improved to a good FC 3 months after the pacemaker implantation, (ii) a poor FC was an independent predictor of both hospitalization and the total mortality, and (iii) the lack of an improvement in the FC at 1 year after the pacemaker implantation was associated with an increased risk of mortality.

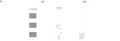

## 1 INTRODUCTION

Patients with bradyarrhythmias undergo ≈1 million de novo pacemaker implantations annually worldwide.^1^ Since the first pacemaker implantation almost 60 years ago, permanent cardiac pacemaker therapy has evolved remarkably, becoming a minimally invasive treatment, improving the quality of life and reducing the mortality.^1^, ^2^, ^3^ Device implantations are now indicated not only for young and middle‐aged individuals who need to maintain physical activity, but also for elderly patients and those with a reduced physical function. There is wide recognition that the functional capacity (FC) in patients with various cardiovascular diseases is an important risk factor for worsening heart failure and an increased risk of mortality,^4^, ^5^ however, there are limited data on whether pacemaker implantations improve the FC, and whether changes in the FC affect the outcomes.

In Japan, there is a system that exempts patients with serious diseases from medical expenses as handicapped disabled patients. In the case of patients with an initial pacemaker implant, the handicapped disability levels can be divided into three levels depending on the indication of the pacemaker implantation defined by the Japanese Circulation Society^6^ and their FC is determined by the metabolic equivalents (METs). Currently, three years after implanting a pacemaker, the disability level is recertified based on the FC at that time. However, it is not known how the FC changes over time after the initial pacemaker implantation, and therefore, the optimal time for the recertification should be determined by prospective studies. Therefore, the aim of this study was to examine the temporal trends in the FC after a pacemaker implantation and the relationship between the FC and prognosis in patients receiving a de novo pacemaker implantation.

## 2 METHODS

### 2.1 Study population

This registry was a prospective, multicenter registry enrolling patient receiving de novo pacemaker implantations at 28 centers in Japan from April 2015 to September 2016. We enrolled patients who were at least 20 years old and had a pacemaker indication according to the Japanese guidelines. We excluded patients who refused to participate in this study. The protocol of this study was approved by the Ethics Committee of the University of Occupational and Environmental Health in Kitakyushu, Japan (H26‐221). Informed consent was obtained from all patients prior to participation, and the study protocol was approved by each institutional Human Investigations Committee. The investigation was performed in accordance with the ethical standards as laid down in the 1964 Declaration of Helsinki and its later amendments.

### 2.2 Data collection and definitions

The patient characteristics and baseline and follow‐up data were obtained through a review of their hospital charts. The anonymized patient data were collected in spreadsheet format by the physicians or clinical research coordinators at each institution. We examined the demographics, etiology of the pacemaker implantation, class of the JCS guideline indication,^6^ and history of heart failure. The history of heart failure was determined as acute heart failure or worsening of chronic heart failure requiring hospitalization. The FC was estimated from the interview of the activities of daily living using a questionnaire^7^ translated into Japanese by the National Institute of Health and Nutrition.^8^ FC was a priori classified into poor FC (<2 METs), moderate FC (2 ≤ METs < 4), and good FC (≥4 METs) (Supplementary file).

### 2.3 Follow‐up and endpoints

After the implantation, the patients were followed‐up at each hospital once every few months for 6 months, and thereafter once every 6 months. The FC was recorded at 3 months, 6 months, 1 year, 2 years, and 3 years after the pacemaker implantation. The endpoints were cardiovascular hospitalization and all‐cause mortality.

### 2.4 Statistical analysis

Continuous variables are expressed as the mean ± SD or median with the interquartile range. Continuous variables were compared with a Student's *t* test. Categorical variables and the distribution of the FCs were analyzed using chi‐squared test. Univariate and multivariate analyses with a Cox proportional hazard regression model was used to identify the significant predictors of the outcomes. The multivariate Cox proportional hazard analysis was adjusted for the age, gender, and significant variables in the univariate analyses, and a history of heart failure and atrial fibrillation. The event‐free curves were computed using the Kaplan‐Meier method and compared with a log‐rank test. A *P* < .05 was considered statistically significant. We used JMP version 11.0 software (SAS Institute Inc, Cary, NC, USA).

## 3 RESULTS

### 3.1 Patient characteristics

All patients underwent a successful pacemaker implantation, and there were no complications or deaths 30 days postprocedure. The baseline characteristics of the patients are shown in Table 1. The mean age was 76 ± 9 years (range 29‐98 years), and there were 315 males (50.7%). The etiology of the device implantation was identified as atrioventricular block in 307 (49.4%) patients, sick sinus syndrome in 276 (44.4%), atrial fibrillation in 32 (5.2%), and others in the remaining six (1.0%). At the time of the device implantation, 583 (93.9%) patients were diagnosed with a grade I disability because of a JCS guideline class I indication. A history of heart failure was noted in 238 (38.3%) patients.

**TABLE 1:** Baseline clinical characteristics

	All patients (n = 621)
Age, years	76 ± 9
Male, n (%)	315 (50.7)
Etiology of pacemaker implant, n (%)	
Atrioventricular block	307 (49.4)
Sick sinus syndrome	276 (44.4)
Atrial fibrillation	32 (5.2)
Others	6 (1.0)
Class of JCS guideline indication, n (%)	
I	583 (93.9)
IIa	36 (5.8)
IIb	2 (0.3)
History of heart failure, n (%)	238 (38.3)
Physical activity, n (%)	
Poor FC	40 (6.4)
Moderate FC	239 (38.5)
Good FC	342 (55.1)

Note

Data are presented as the mean ± SD or number (%) of patients. Poor FC was defined as < 2 METs, moderate FC as 2 ≤ METs<4, and good FC as ≥ 4 METs.

Abbreviations: JCS, Japanese Circulation Society; FC, functional capacity; METs, metabolic equivalents.

### 3.2 Changes in the functional capacity

**FIGURE 1:**
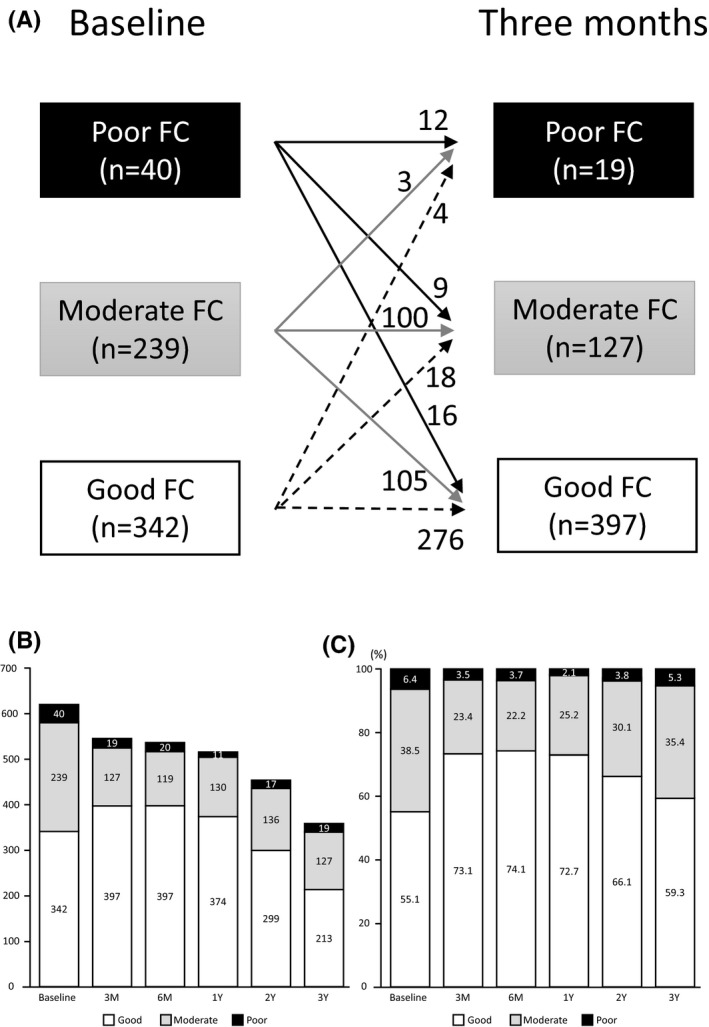
The time course of the functional capacity (FC). (A) Changes in the FC between baseline and 3 months after the pacemaker implantation. Three months after the pacemaker implantation, 43% of those with poor and moderate FC at baseline improved to good FC (16 + 105/40 + 239). During the 3 months follow‐up period, 78 patients were lost because they returned to the hospital or clinic that had been referred after the device implantation. (B) The raw number of FCs during follow‐up. (C) The proportion of three classes of FCs. The patients were a priori classified into poor FC (<2 METs), moderate FC (2 ≤ METs <4), and good FC (≥4 METs). FC = functional capacity, F/U = follow‐up, METs = metabolic equivalents

Figure 1 shows the serial changes in raw numbers and proportions for three classes of FCs. Three months after the pacemaker implantation, 16 patients with poor FC and 105 with moderate FC at baseline improved to good FC (Figure 1A). However, three patients with moderate FC and four with good FC at baseline deteriorated to poor FC at 3 months after the implantation. During the 3 months follow‐up period, 78 patients were lost because they returned to the hospital or clinic that had been referred after the device implantation. Three months after the pacemaker implantation, the distribution of the three FCs remained at those levels until after 1‐year follow‐up (*P* = .18). The rate of poor FC was the lowest (2.1%) in the first year after implantation and tended to increase over time (Figure 1B and C).

### 3.3 Predictors of cardiovascular hospitalizations and all‐cause mortality

**FIGURE 2:**
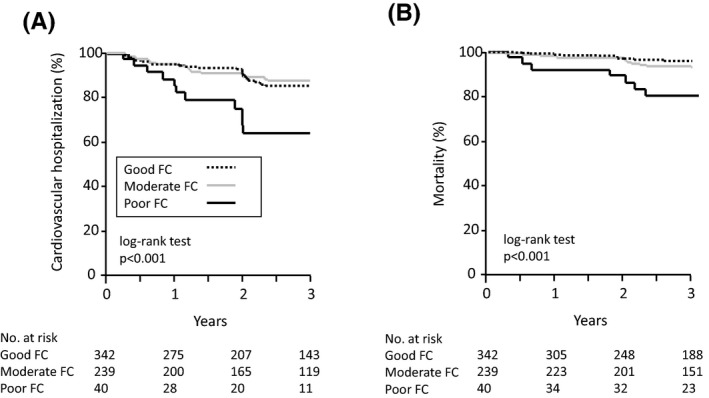
Kaplan‐Meier analysis of the time to the subsequent outcome. (A) The cardiovascular hospitalization rate was higher in the patients with poor FC than in those with moderate or good FC. (B) The total mortality rate was higher in the patients with poor FC than in those with moderate FC or good FC. Abbreviations are shown in Figure 1

During a median of 2.4 years of follow‐up (interquartile range 0.2‐3 years), 71 (11%) patients had a cardiovascular hospitalization (heart failure [n = 45], ischemic heart disease [n = 18], and strokes [n = 8]). A total of 35 patients (5.6%) died as a result of malignancy (n = 8), sepsis (n = 7), heart failure (n = 6), respiratory failure (n = 3), sudden death (n = 2), stroke (n = 1), or other causes (n = 8). The univariate Cox analysis revealed that a history of heart failure and poor FC were significantly associated with a hospitalization (Table 2). The multivariate Cox analysis adjusted for the age and gender, history of heart failure, and poor FC at baseline revealed that a history of heart failure (HR 2.097, 95% CI 1.275‐3.448, *P* = .004) and poor FC at baseline (HR 2.494, 95% CI 1.227‐5.070, *P* = .012) remained as independent predictors. For the all‐cause death, the age (HR 1.096, 95% CI 1.043‐1.151, *P* < .001) and poor FC at baseline (HR 3.338, 95% CI 1.254‐8.886, *P* = .016) were independent predictors after being adjusted for the age and gender, and poor FC at baseline (Table 3). The Kaplan‐Meier analysis demonstrated that the rate of a hospitalization (Figure 2A) and the total mortality were significantly higher as the FC decreased (Figure 2B).

**TABLE 2:** Results of the univariate and multivariate analyses for the prediction of a hospitalization

Variable	Univariate analysis			Multivariate analysis		
	HR	95% CI	*P* value	HR	95% CI	*P* value
Age, per 1‐year increase	1.003	0.978‐1.028	0.820	0.996	0.971‐1.021	0.744
Male gender	1.308	0.818‐2.091	0.262	1.446	0.885‐2.363	0.141
Etiology of pacemaker implant						
Atrioventricular block	0.930	0.584‐1.481	0.759			
Sick sinus syndrome	0.813	0.505‐1.309	0.394			
Atrial fibrillation	2.092	0.958‐4.566	0.064	1.555	0.701‐3.449	0.278
History of heart failure	2.189	1.367‐3.505	0.001	2.097	1.275‐3.448	0.004
Physical activity						
Poor FC vs. Good FC	3.078	1.566‐6.050	0.001	2.494	1.227‐5.070	0.012
Moderate FC vs. Good FC	0.880	0.525‐1.475	0.628			

Abbreviations: HR, hazard ratio; CI, confidence interval; Other abbreviations are as in Table 1.

**TABLE 3:** Results of the univariate and multivariate analyses for the prediction of the all‐cause mortality

Variable	Univariate analysis			Multivariate analysis		
	HR	95% CI	*P* value	HR	95% CI	*P* value
Age, per 1‐year increase	1.105	1.052‐1.159	<0.001	1.096	1.043‐1.151	<0.001
Male gender	1.867	0.929‐3.752	0.796	2.411	1.177‐4.937	0.161
Etiology of pacemaker implant						
Atrioventricular block	0.686	0.349‐1.348	0.274			
Sick sinus syndrome	1.042	0.536‐2.027	0.902			
Atrial fibrillation	1.797	0.550‐5.871	0.332	2.184	0.641‐7.448	0.212
History of heart failure	0.921	0.464‐1.828	0.814	0.673	0.327‐1.386	0.283
Physical activity						
Poor FC vs. Good FC	4.846	1.907‐12.311	<0.001	3.338	1.254‐8.886	0.016
Moderate FC vs. Good FC	1.726	0.816‐3.650	0.153			

Abbreviations: HR, hazard ratio; CI, confidence interval. Other abbreviations are as in Table 1.

### 3.4 Subgroup analysis of poor functional capacity patients 1 year after the pacemaker implantation

**FIGURE 3:**
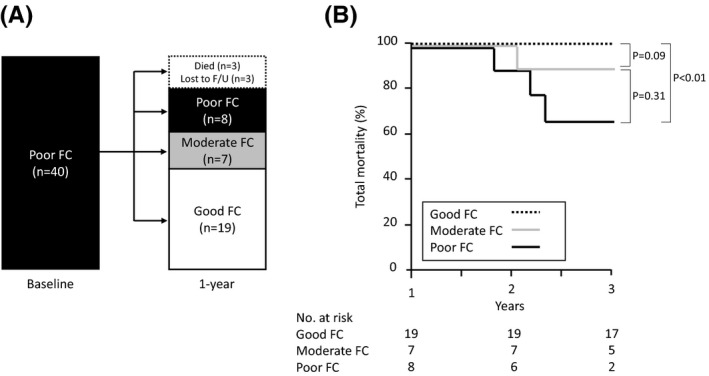
Subgroup analysis among the patients with poor functional capacity. (A) Changes in the functional capacity during 1‐year follow‐up. Seven (17.5%) patients improved to moderate FC, and 19 (47.5%) had a marked improvement. Abbreviations are shown in Figure 1. (B) The total mortality rates. A log‐rank test was not applied because no patients with good FC died

We further examined the outcome of the 40 patients with poor FC at the time of the pacemaker implantation (Figure 3A). Three patients died and three were lost to follow‐up at 1 year after the device implantation. Of the remaining 34 patients, eight (20%) had no improvement in the FC, but seven and 19 patients improved to moderate FC and good FC, respectively. Those 34 patients were further followed for a median of 2 years (interquartile range 0.3‐2 years). The patients who remained with poor FC one year after the pacemaker implantation had a significantly higher mortality rate (37.5%) than those that improved to good FC (Figure 3B).

## 4 DISCUSSION

We examined the temporal trends in the FC and the relationship between the FC and prognosis in patients receiving an initial pacemaker implantation. The major findings of this study were that (i) 43% of the patients with a poor or moderate FC improved to good FC 3 months after the pacemaker implantation, (ii) poor FC was an independent predictor of both hospitalization and total mortality, and (iii) the lack of an improvement in the FC at 1 year after the pacemaker implantation was associated with an increased risk of mortality.

It is well established that the exercise capacity, daily physical activity, and daily walking performance are significantly associated with the clinical outcome.^9^, ^10^, ^11^ Recent studies using pacemaker built‐in accelerometers found a significant correlation between physical activity and mortality. Tyagi et al assessed the physical activity measured by a pacemaker accelerometer in de novo pacemaker implantation patients who had a preserved left ventricular function.^12^ They classified the patients into four groups based on the average active time and followed them for an average of 4.1 years. The rate of the all‐cause mortality significantly increased as the active minutes decreased. Patients with an average of <1 h/day of active time had a nearly 7.5‐fold increased risk of death compared to those who were active >3 h/day. In another study, Conraads et al showed a significant relationship between the physical activity and the mortality in patients with implantable cardioverter defibrillators (ICDs) and reduced the left ventricular ejection fraction. The patients in the lowest tertile for daily activity (<146 min/day) had five times higher risk of mortality compared to those in the highest tertile for daily activity (>235 min/day).^13^ These data suggested that the device‐measured physical activity may have served as a marker for the unmeasured factors contributing to the mortality risk. In addition, a novel chronotropic incompetence measure (Heart Rate Score) also predicts a worse outcome in patients who undergo device implantations.^14^, ^15^ Furthermore, Richards et al suggested that a blended sensor with minute ventilation and an accelerometer improves the Heart Rate Score in patients with pacemakers.^16^ While these device built‐in objective indicators may be useful to quantify an individual's activity, this device software has difficulty in interpreting because the measurement method and calculation method differ depending on the manufacturer. Additionally, device interrogation or remote monitoring are required to use these indicators. On the other hand, a subjective FC by a questionnaire, not a device‐measured physical activity, is easily obtainable and is able to predict the outcome.

In this study we showed that 43% of the patients with poor or moderate FC at the time of the pacemaker implantation had improved FC 3 months after the implantation and improved FC was associated with a better outcome. Previous studies showed that the device‐measured physical activity increased over a 30‐day period after the implantation in patients that received an ICD or cardiac resynchronization therapy defibrillator.^13^ That observation was consistent with our study, in that most of the patients had improved FC probably because the bradycardia‐associated symptoms were alleviated by the pacemaker. Fleischmann et al demonstrated that the pacemaker implantation itself was associated with a significant improvement in the health‐related quality of life (QOL) scores. This improvement extended to almost all domains such as the physical function, physical role, social function, mental health, and vitality.^17^ Importantly, a QOL improvement was similarly observed irrespective of the gender, presence of heart failure, or comorbidity level. We suspected that the improved QOL with the pacemaker implantation may also have influenced the subsequent prognosis in this study.

To preserve or improve the physical activity after the permanent pacemaker implantation, physiological pacing is expected to be a promising strategy. Minimizing any inadvertent ventricular pacing is important for maintaining the exercise capacity and preventing subsequent cardiac events. A more physiological pacing such as His‐bundle pacing^18^ is a promising option. Among patients with left ventricular (LV) systolic dysfunction and a wide QRS complex or with ventricular pacing dependency, cardiac resynchronization therapy may be a better device to improve the exercise tolerance. An improvement in the hemodynamic profile and sympatho‐inhibitory effect lead to a reversal of skeletal myopathy and an enhanced exercise performance. Cardiac resynchronization increases the LV contractility and reduces functional mitral regurgitation, resulting in an increased cardiac output and dp/dt index. These improvements in the cardiac hemodynamics result in a decrease in the muscle sympathetic nerve activity, reversal of muscle inflammation, and improve the long‐term skeletal myopathy.^19^

Our study showed that physical inactivity was associated with a poor survival independent of other risk factors. Cardiologists should pay attention not only to the device condition but also to encourage increased physical activity and to follow the patient compliance with physical activity recommendations. Considering the association between significantly reduced physical activity and a poor prognosis, individuals with an FC of <2 METs were stratified into serious conditions and required careful observation. In the majority of patients, the FC improved at 3 months after the pacemaker implantation and was maintained for at least 1 year. Patients with an improved FC at 1 year after the pacemaker implantation had a relatively good prognosis. Particularly, no patients died in the group with an improved FC (METs ≥ 4). Thus, a reevaluation of the physical disability between 3 months and one year after implantation gives the lowest percentage of poor FC.

### 4.1 Study limitation

Our study included heterogenous patients receiving pacemakers for atrioventricular block, sick sinus syndrome, and atrial fibrillation with a slow ventricular response. We did not investigate the association between atrial or ventricular pacing frequency and prognosis. We did not investigate the detailed pacemaker pacing mode, pacing rate, heart rate distribution (ie, Heart Rate Score), or pacing site, therefore, the relationship between the pacemaker settings and physical activity could not be examined. However, irrespective of the device status, poor FC was proven to be useful as a prognostic indicator. The conventional prognostic clinical tests such as the B‐type natriuretic peptide, renal function, echocardiographic findings, and cardiopulmonary exercise testing were not analyzed. This study did not include the currently available leadless pacemakers.

## 5 CONCLUSION

The pacemaker implantation improved the FC in 43% of the patients with a poor or moderate FC at baseline and remained at that level to the end of 1 year. The poor FC (<2 METs) at baseline was significantly associated with a worse outcome. The patients whose FC improved at 1 year after the pacemaker implantation had a relatively good prognosis.

## CONFLICTS OF INTEREST

EW received a lecture fee from Biotronik Japan, Daiichi‐Sankyo, and Pfizer. RK has an affiliation with the endowed departments of Boston Scientific and Abbott. AN received a lecture fee from Daiichi‐Sankyo and has an affiliation with the endowed department of Boston Scientific and Abbott. KO received a lecture fee from Johnson and Johnson, Medtronic, Daiichi‐Sankyo, and Boehringer‐Ingelheim. None of the other authors have conflicts of interest.

## Supporting information

Table S1
