# Supplementary material for: Impact of a poor functional capacity on the clinical outcomes in patients with a pacemaker implantation –Results from the Japanese Heart Rhythm Society Registry –
Source: J Arrhythm. 2020 Dec 9;37(1):182–8. doi: 10.1002/joa3.12459 (PMC7896460; doi:10.1002/joa3.12459)
Supplement: Supplementary file 1 — Table S1 [file JOA3-37-182-s001.docx]

**Supplementary files**

**Table**. Grades of the physical disability at the time of the initial pacemaker implantation.^1^

| Grade | Details |
| --- | --- |
| Grade 1 | Class 1 indication, or  Class 2 indication and a functional capacity of <2 METs |
| Grade 2 | Class 2 indication and a functional capacity of 2< METs <4 |
| Grade 3 | Class 2 indication and a functional capacity of >4 METs |

In the case of a *de novo* implantation, if the indication is a guideline class 1,^2^ it is certified as a Grade 1 regardless of the functional capacity. Three years after the implantation, a recertification will be performed only by the physical activity as follows: Grade 1 (<2 METs), Grade 2 (2< METs <4), and Grade 3 (>4 METs). Patients with a pacemaker implantation under the age of 18 due to congenital heart disease are rated as Grade 1.

**References**

**1.** Cardiac dysfunction (pacemaker implantation) and limb disability (artificial joint replacement) disability certification standards. Available at: https://www.mhlw.go.jp/public/bosyuu/iken/p20131125-03.html. Accessed April 12, 2020.

**2.** 2018 JCS/JHRS Guideline on Non-Pharmacotherapy of Cardiac Arrhythmias. Available at: https://www.j-circ.or.jp/cms/wp-content/uploads/2018/07/JCS2018_kurita_nogami191120.pdf. Accessed April 12, 2020.
